# Promoting human rights in mental healthcare: beyond the ‘Geneva impasse’

**DOI:** 10.1192/bjo.2023.546

**Published:** 2023-08-11

**Authors:** Jill Stavert, George Szmukler

**Affiliations:** Centre for Mental Health Practice, Policy and Law Research/Centre for Mental Health and Capacity Law, School of Health and Social Care, Edinburgh Napier University, Edinburgh, UK; Institute of Psychiatry, Psychology and Neuroscience, King's College London, London, UK

**Keywords:** Human rights, psychiatry and law, autonomy, consent and capacity, ethics

## Abstract

The past decade has seen a significant growth in attention to the human rights of persons with disabilities, taken to include mental health conditions. Consequently, challenges to important areas of current psychiatric practice have emerged, with which the profession has, in general, shown limited engagement.

If a person with mental disability is to live the best life they can, on their own terms, the full range of their human rights must be recognised, as these underpin the enablement and necessary support required to achieve this. The United Nations (UN) Convention on the Rights of Persons with Disabilities (CRPD)^1^ details those rights. Its model is that the degree to which a physical or mental impairment is a disability depends on the extent to which society makes reasonable accommodations to the impairment. A person's diagnosis or related impairment must not justify inequalities in the enjoyment of those rights.

Ensuring and enabling individual autonomy on an equal basis with others is essential in the context of psychiatric care and treatment. Human rights are interconnected; the full range of civil, political, economic, social and cultural rights must be considered. We agree with McSherry et al^2^ that the polarising debate around autonomy has detracted from giving effect to the CRPD at national level and we support a broader-based approach to human rights in mental health law.

The ‘Geneva impasse’ refers to the disagreement between a number of UN bodies about whether the right to ‘legal capacity’ requires the abolition of all forms of involuntary treatment.^3^ Such disagreement extends far beyond the UN itself, including virtually all of the stakeholders in mental healthcare and policy and their representative bodies.

## Scottish Mental Health Law Review

The need to address how to give effect to the CRPD and tackle the ‘Geneva impasse’ was an important consideration for the Scottish Mental Health Law Review (Scott Review), which commenced in May 2019 and reported in September 2022.^4^ Its overall remit was: ‘ … to improve the rights and protections of persons who may be subject to the existing provisions of mental health, incapacity or adult support and protection legislation’. CRPD rights are not at present legally enforceable in Scotland, but the Scottish Government has indicated a commitment to give these rights legal effect, along with those in other international human rights treaties, although the content and effect of the awaited legislation is not yet known. Meanwhile, the devolution arrangements between Scotland and the UK require that proposed devolved legislation and Scottish Ministers’ actions do not place the UK in violation of the CRPD.

The Scott Review was the result of increasing stakeholder concern that existing mental health and capacity law inadequately support the needs of persons who the legislation was designed to help and have become misaligned with developing human rights standards, notably those of the CRPD.^5^ The current legislation, still largely European Convention on Human Rights compliant, focuses on civil rights that safeguard persons with ‘mental disorder’ from unwarranted interventions. It gives insufficient priority to a person's will and preferences and fails to address those rights (predominantly economic, social and cultural rights) that are essential to achieving the highest attainable standard of mental health and for living independently.

After engaging and consulting widely with stakeholders, including persons with mental illness and their families/carers and psychiatrists, the Review identified three key themes that need to be reflected in mental health and capacity law and practice:

- strengthening the voice of people who use services and those who care for them
- reducing the need for coercion in the system
- securing rights to the help and support needed to live a good life.

The Review reflected these in its final report, which recommended that this would require a repurposing of mental health and capacity law away from a focus on the authorisation and regulation functions to also ensuring and enabling the meeting of needs at the right time. This would take place within a framework that encompasses:

- human rights enablement (HRE)
- supported decision-making (SDM), which ensures that the person's voice is heard at all times on an equal basis with others
- autonomous decision-making (ADM).

The recommended HRE and ADM approaches provide only the theoretical framework. Their operational practicalities must be clarified and they must be accompanied by a robust accountability and scrutiny framework based on data that people with mental disabilities can understand. Whether they will be located in broader human rights legislation or in mental health and capacity legislation remains to be decided. However, the objective of HRE is to provide a mechanism whereby the whole range of an individual's applicable rights are identified, balanced and enabled in any situation to ensure their needs and wishes are non-discriminatorily met. The ADM recognises that sometimes, for reasons that might or might not include diagnosis, a person is unable to make, communicate and put into effect their autonomous will and preferences. In such cases, non-consensual measures will only be used where they are necessary to protect the person's rights and their needs and autonomous wishes cannot be met by other measures.

Although evidence suggested that total abolition of compulsory psychiatric treatment, as required by the Committee on the Rights of Persons with Disabilities (CRPD Committee),^6^ was not immediately achievable (and perhaps would never entirely be), the Review considered that there should be an initiative across law, policy and practice to significantly reduce the use of compulsory psychiatric measures. In this it was following the approach of the former UN Special Rapporteur on the Right to Health, which also reinforced the need to be able to offer a range of alternatives to non-consensual measures.^7^

## Psychiatrists and reform

The limited engagement of psychiatrists and their professional organisations with current international movements to re-examine the place of human rights in mental healthcare is disappointing. The 2014 CRPD Committee's interpretation of Article 12 (General Comment No. 1)^6^ when presented as a draft for public consultation received no submission from any national or international representative psychiatry organisation (with the possible exception of the Human Rights Committee of the World Association for Psychosocial Rehabilitation), despite its hugely controversial interpretation that ‘substitute decision-making’ is in violation of the Convention. Reference to the CRPD on psychiatry professional bodies’ websites are rare.

A strong case has been made that conventional mental health laws discriminate unfairly against people with a diagnosis of a mental disorder.^8^ They give expression to two prejudicial, deeply culturally rooted stereotypes – that those persons are incapable of making rational, sound judgements and that they are intrinsically dangerous. A limited engagement by psychiatrists with national rights-based mental health law reform is evident – although the Scott Review, unusually, succeeded in establishing substantial dialogue with the Scotland branch of the Royal College of Psychiatrists. Equally disappointing is psychiatrists’ relative lack of interest in growing international concern about coercive interventions, especially when one sees a 20-fold variation in the rate per 100 000 population of involuntary hospital admissions across Western European nations.^9^ Yet despite a strong position statement and call for action from the World Psychiatric Association^10^ (as well as a resolution, no. 2291, adopted in 2019 by the Council of Europe – ‘Ending coercion in mental health’^11^) there has so far been little reaction from professional bodies. Research has been meagre – only 42 studies (in English) between 1990 and 2018 on measures designed primarily to reduce coercion.^12^

Medical practitioners are generally wary of rushing to adopt new measures that may turn out to be ineffective or indeed harmful. Psychiatrists particularly so, because of their regular exposure to varying accounts of the putative nature of mental health conditions, their determinants and their treatment, as regularly offered by key society stakeholders, especially service user groups. Psychiatrists sit nearer a ‘paternalism’ pole, whereas reform movements sit nearer an ‘autonomy’ pole on a conceptual spectrum joining the two. In the former, clinical matters largely set the standard for right action. In the latter, a central aim is to establish a fuller account, including the person's deeply held beliefs and values and their particular social context. Different kinds of evidence thus are adduced – the former leans on the scientific characterisation of the condition, evidence-based treatment and a prudent choice for a clinically desirable outcome; the latter leans on the person's beliefs and values as expressed, for example, in an advance choice document or ascertained through discussion with the person and those who know the person well, and seeks an outcome that gives effect as far as possible to the person's important life projects.

A timely shift in practice towards the ‘autonomy’ pole may move us towards opening the general, quotidian ‘impasse’. One of us, for example, has proposed a reformulation of decision-making ability and ‘best interests’ in terms of ‘will’ and ‘preferences’.^13^ Furthermore, Ruck Keene et al^14^ detect a possible ‘softening’ of the CRPD Committee's injunction on ‘substitute decision-making’. The CRPD Committee's 2019 ‘concluding observations’ state that Australia should ‘implement a nationally consistent supported decision-making framework, as recommended in a 2014 report of the Australian Law Reform Commission entitled *Equality, Capacity and Disability in Commonwealth Laws*’.^15^ But, as Ruck Keene et al note, the Australian Law Reform Commission's report states (at para. 3.48):

‘with appropriate safeguards, and a rights emphasis, there is no “discriminatory denial of legal capacity” necessarily inherent in a functional test [of decision-making capacity or ‘ability’] – provided the emphasis is placed principally on the support necessary for decision-making and that any appointment [of a decision-maker] is for the purpose of protecting the person's human rights’.^16^

The Scott Review's recommendations further develop such a framework.

## Data Availability

Data availability is not applicable to this article as no new data were created or analysed in this study.
